# Flow cytometry analysis of immune and glial cells in a trigeminal neuralgia rat model

**DOI:** 10.1038/s41598-021-02911-x

**Published:** 2021-12-07

**Authors:** Junjin Lin, Luxi Zhou, Zhaoke Luo, Madeha Ishag Adam, Li Zhao, Feng Wang, Daoshu Luo

**Affiliations:** 1 Public Technology Service Center of Fujian Medical University; Laboratory of Clinical Applied Anatomy, School of Basic Medical Sciences, Fujian Medical University, Fuzhou, 350122 P.R. China; 2 Key Laboratory of Brain Aging and Neurodegenerative Diseases of Fujian Province, Fuzhou, 350122 P.R. China; 3 Department of Human Anatomy, School of Basic Medical Sciences, Fujian Medical University, No. 1 Xuefu North Road, University Town, Fuzhou, 350122 P.R. China

**Keywords:** Neuroimmunology, Sensory processing, Nervous system

## Abstract

Microvascular compression of the trigeminal root entry zone (TREZ) is the main cause of most primary trigeminal neuralgia (TN), change of glial plasticity was previously studied in the TREZ of TN rat model induced by chronic compression. To better understand the role of astrocytes and immune cells in the TREZ, different cell markers including glial fibrillary acidic protein (GFAP), complement C3, S100A10, CD45, CD11b, glutamate-aspartate transporter (GLAST), Iba-1 and TMEM119 were used in the TN rat model by immunohistochemistry and flow cytometry. On the post operation day 28, GFAP/C3-positive A1 astrocytes and GFAP/S100A10-positive A2 astrocytes were activated in the TREZ after compression injury, there were no statistical differences in the ratios of A1/A2 astrocytes between the sham and TN groups. There was no significant difference in Iba-1-positive cells between the two groups. The ratios of infiltrating lymphocytes (CD45+CD11b−) (p = 0.0075) and infiltrating macrophages (CD45highCD11b+) (p = 0.0388) were significantly higher than those of the sham group. In conclusion, different subtypes A1/A2 astrocytes in the TREZ were activated after compression injury, infiltrating macrophages and lymphocytes increased, these neuroimmune cells in the TREZ may participate in the pathogenesis of TN rat model.

## Introduction

Primary trigeminal neuralgia (TN) is a common clinical paroxysmal and severe chronic neuropathic pain. Microvascular compression of the trigeminal root entry zone (TREZ) is considered to be the main cause of most primary TN patients^1^. However, the pathogenesis of TN is still unclear. The TREZ is a transitional nerve root where both central nervous system (CNS) and peripheral nervous system (PNS) glial cells participate in the CNS-PNS interface^2,3^. There are abundant astrocytes and myelinating oligodendrocytes in the central portion of the TREZ and myelinating and nonmyelinating Schwann cells in the peripheral portion. It was reported that astrocytes in the central portion of the TREZ were activated after compression injury in TN rats; however, there was no significant change in the morphology of oligodendrocytes^4^. Iba-1-immunoreactive microglia/macrophages in the TREZ also activated after compression injury in TN rats^4^.

Both microglia and macrophages are involved in the development of neuropathic pain^5^. Astrocyte crosstalk with microglia plays a key role in the neuroimmune response and the development of neuropathic pain^6^. Activated astrocytes synthesize and secrete granulocyte–macrophage colony stimulating factor (GM-CSF) to regulate the transcription of inflammation-related genes in microglia and infiltrating monocytes in the CNS^7^. It was reported that the structure and function of activated astrocytes under different conditions were different, and reactive astrocytes could be divided into A1-type neurotoxic astrocytes and A2-type neuroprotective astrocytes^8^. Whether these subtypes of astrocytes also participate in the pathogenesis of TN is still unknown.

Our previous studies observed that chronic compression of the TREZ induced local plasticity changes in a variety of glial cells and protrusions of reactive astrocytes extending from the CNS side to the PNS side^4^, but we did not distinguish microglia from infiltrating macrophages or different subtypes of astrocytes. Astrocytic glutamate-aspartate transporter (GLAST) is a well-known marker of mature astrocytes and complement C3 and S100 calcium-binding protein A10 (S100A10) are also identified as markers of A1 astrocytes and A2 astrocytes, respectively^8–10^. Furthermore, microglia and infiltrating macrophages in the nervous system have some similar lineage characteristics, including origin, proliferation and the expression of myeloid cell markers; therefore, it was difficult to distinguish the microglia and infiltrating macrophages in the TREZ by Iba-1 immunostaining. It was reported that detecting the differential expression of CD11b and CD45 in microglia and macrophages by flow cytometry was a good way to distinguish them^11,12^.

Therefore, different cell markers were used in this study to further clarify the categories of microglia, infiltrating macrophages, lymphocytes and astrocytes activated in the TREZ after compression injury in the TN rat model by immunohistochemistry and flow cytometry, which provide an experimental basis for further studying the pathogenesis of TN.

## Materials and methods

### Animal

Healthy adult male Sprague–Dawley rats weighing 150 ± 20 g were obtained from the Experimental Animal Center of Fujian Medical University. The rats were housed under a 12:12 h light/dark cycle at a constant room temperature. All experiments were approved by the *Laboratory Animal Management and Use Committee of Fujian Medical University* and were performed in accordance with the *International Association for the Study of Pain (IASP)* rules on the use of experimental animals to minimize the number of animals used. Animals studies were performed in accordance with ARRIVE guidelines.

### TN rat model of chronic compression in TREZ

The experimental animals were randomly divided into a sham operation group and a TN group (n = 4–5 for each group) with chronic compression in the TREZ. The TN animal model was established according to our previously described procedure^13,14^. A surgical incision was made at the upper edge of the right orbit of the rat. Then, the infraorbital fissure was exposed along the operative approach of the medial wall of the orbit, and a small plastic filament was carefully inserted into the intracalvarium from the inferior orbital fissure to compress the TREZ. In the sham group, the right infraorbital nerve of rats was only surgically exposed, without TREZ compression injury.

### Measurement of the orofacial mechanical pain threshold

von Frey filaments were used to measure the orofacial mechanical pain threshold of the rats. The right orofacial area was stimulated by a von Frey filament 3 times with an interval of 10 s. The filament was bent during stimulation, and the strength of the filament was recorded when positive reactions of orofacial pain behaviors were observed. The mechanical pain thresholds of rats in each group were recorded 3 days before surgery to obtain baseline data and were measured again on postoperative day (POD) 28 to evaluate the mechanical allodynia of the TN animal model.

### Immunohistochemistry

Rats in both groups were deeply anesthetized with sodium pentobarbital (200 mg/kg, i.p.) on POD 28 and then perfused through the left ventricle with 4% paraformaldehyde phosphate buffer (pH 7.4). The TREZ segment was dissected and then cryoprotected with 30% (W/V) sucrose in 0.1 M PBS overnight at 4 °C. A series of 10-μm sections of trigeminal nerve roots were longitudinally sectioned with a Cryostat Microtome (Leica CM1950, Heidelberger, Germany).

For immunohistochemical staining, sections were washed three times in 0.1 M phosphate-buffered saline (PBS) for 10 min and blocked in 3% bovine serum albumin (BSA) for 30 min. The sections were subsequently incubated with primary antibodies after removal of the blocking solution without washing them. The following primary antibodies were incubated with the sections for 24 h at 4 °C: rabbit monoclonal antibody against Ki67 (1:250, Abcam, ab16667, Cambridge, UK), mouse monoclonal antibody against C3 (1:100, Santa Cruz, sc-28294, Texas, USA), chicken polyclonal antibody against S100A10 (1:100, Abcam, ab50737, Cambridge, UK), rabbit polyclonal antibody against glial fibrillary acidic protein (GFAP) (1:1000, Proteintech, 16825-1-AP, IL, USA), mouse monoclonal antibody against GFAP with Cy3 conjugated (1:1000, Sigma, MAB3402C3, MO, USA), goat polyclonal antibody against Iba-1 (1:200, Novus, NB100-1028, CO, USA) and mouse monoclonal antibody against TMEM119 (1:500, Proteintech, 66948-1-Ig, IL, USA). After rinsing with 0.01 M PBS, the slices were further incubated with different secondary antibodies: Alexa Fluor 488-conjugated donkey anti-rabbit IgG (1:1000, Invitrogen, A-21206, CA, USA), Alexa Fluor 647-conjugated goat anti-chicken IgY (1:1000, Invitrogen, A-21449, CA, USA), Alexa Fluor 647-conjugated donkey anti-rabbit IgG (1:1000, Invitrogen, A-32795, CA, USA), biotinylated goat anti-mouse IgG (1:200, Vector, BA-9200, CA, USA), or Cy^TM^3-conjugated streptavidin (1:500, Jackson ImmunoResearch, 016-160-084, PA, USA). DAPI (1:1000, Beyotime, C1002, Shanghai, China) was used to stain the nuclei. The immunohistochemical sections were photographed and analyzed with a Leica laser confocal microscope (Leica TCS SP8, Heidelberger, Germany). Image-Pro Plus software (version 6.0; Media Cybernetics, MD, USA) also helped to quantify and graph positive labelling in a specific cell type (i.e. C3 expression in GFAP + astrocytes) as area fraction, which is the percentage of double positive labelling in the cell type of interest within the imaged area, as previously described^15,16^.

### Flow cytometric analysis

TREZ and TG tissues were harvested at POD 28 in both groups. A neural tissue dissociation kit (Miltenyi Biotec, Bergisch Gladbach, Germany) and Gentle MACS™ dissociators (Miltenyi Biotec, Bergisch Gladbach, Germany) were used to obtain single cells. Thereafter, 1 µL of Golgi transport protein blocker GolgiPlug Protein Trnsp Inhb (BD Pharmingen, NJ, USA) was added to the 10^6^/mL cell culture medium. Then, the cells were cultured at 37 ℃ in a 5% CO_2_ incubator for 6 h. Fixable Viability Stain 780 (BD Pharmingen, NJ, USA) was used to mark dead cells.

In each tube, to 100 μL of liquid, 2 µL of FC receptor blocker CD32 (550270, BD Pharmingen, NJ, USA) was added and incubated at room temperature for 5 min in the dark, followed by the addition of 2 µL of flow cytometry antibodies CD45-PE-Cy7 and CD11b-V450 (561588 and 562108, BD Pharmingen, NJ, USA), GLAST (130-118-344, Miltenyi Biotec, Bergisch Gladbach, Germany) and incubating at room temperature for 15 min in the dark. After incubation, the suspended cells were centrifuged at 300 × *g* for 5 min, and then the supernatant was discarded.

A volume of 100 µL of fixation/permeabilization solution (BD Pharmingen, NJ, USA) was added to each tube and incubated at 4 °C for 20 min in the dark. Then, 1 ml of 1 × Perm/Wash Buffer (BD Pharmingen, NJ, USA) was added, and the cells were washed twice by centrifugation at 300 × *g* for 5 min. After resuspension in 100 µL of 1 × Perm/Wash buffer, the cells were incubated with 0.2 μg/mL anti-S100A10-Alexa Fluor 647 antibody (FAB2377R, R&D Systems, MN, USA) and 0.1 μg/mL anti-C3 antibody (ab17456, Abcam, Cambridge, UK) at room temperature for 30 min. Then, the cells were centrifuged at 400 × *g* for 5 min and resuspended in ice-cold PBS. Fluorescent dye-labeled secondary antibody (A11001, Thermo, MA, USA) was added and incubated for 20 min at room temperature. Finally, the cells were washed and transferred to Trucount™ Absolute Counting Tubes for flow cytometric analysis. Data were acquired on a BD LSR Fortessa X-20 (BD Biosciences, NJ, USA) and analyzed with FlowJo V10 (BD Pharmingen, NJ, USA).

### Statistical analysis

All data are presented as the mean ± standard error of the mean (SEM). Data analysis of the flow cytometry plots was performed using FlowJo V10 (BD Pharmingen, NJ, USA). Data were analyzed using SPSS 19.0 statistical software (IBM, NK, USA) and GraphPad Prism 8 software (GraphPad Software, CA, USA). Statistical differences between groups of rats were calculated using two-way ANOVA with Sidak’s multiple comparisons tests and Student’s t-tests. *p* < *0.05* was considered statistically significant.

## Results

### Chronic compression of the TREZ induced orofacial mechanical hyperalgesia in TN rats

**Figure 1:**
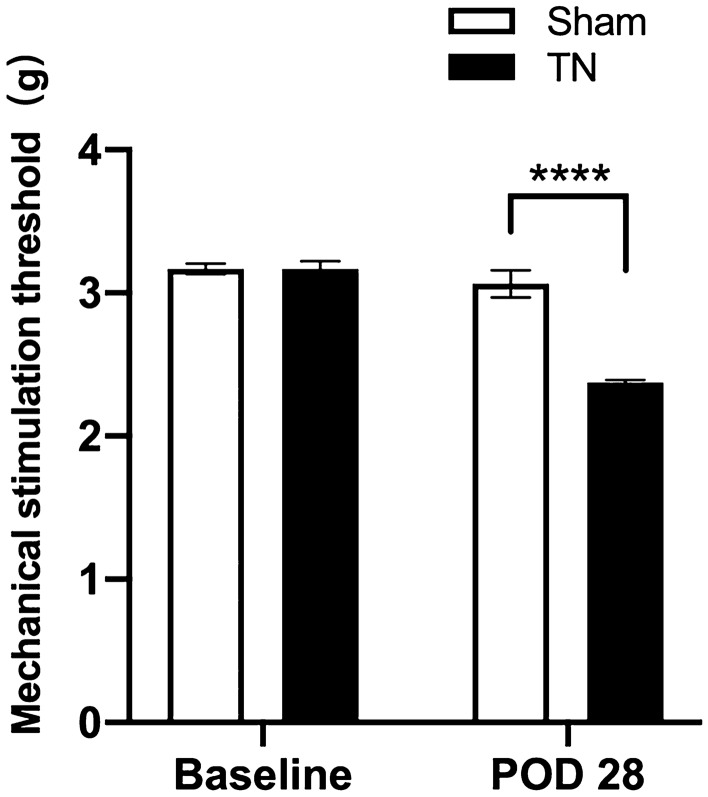
Orofacial mechanical stimulation threshold changes in the TN rat model. There was no significant difference in the hyperalgesia threshold of the right orofacial mechanical stimulation between the TN group and the sham group (n = 4 for each group) before surgery. On the 28th day after the operation, the mechanical stimulation threshold in the sham group returned to the preoperative baseline threshold, while the mechanical stimulation threshold of rats in the TN group was significantly lower than that of the sham group and at baseline. ****p < 0.0001 as determined by two-way ANOVA with Sidak’s multiple comparisons tests. All data show the mean ± SEM.

There was no significant difference in the baseline pain threshold between the sham group and the TN group. However, the mechanical stimulation hyperalgesia threshold of the TN group was significantly lower than that of the sham group on POD 28 (Fig. 1), indicating that the TN animal model was successfully established.

### Different types of astrocytes were activated in the TREZ after compression injury

**Figure 2:**
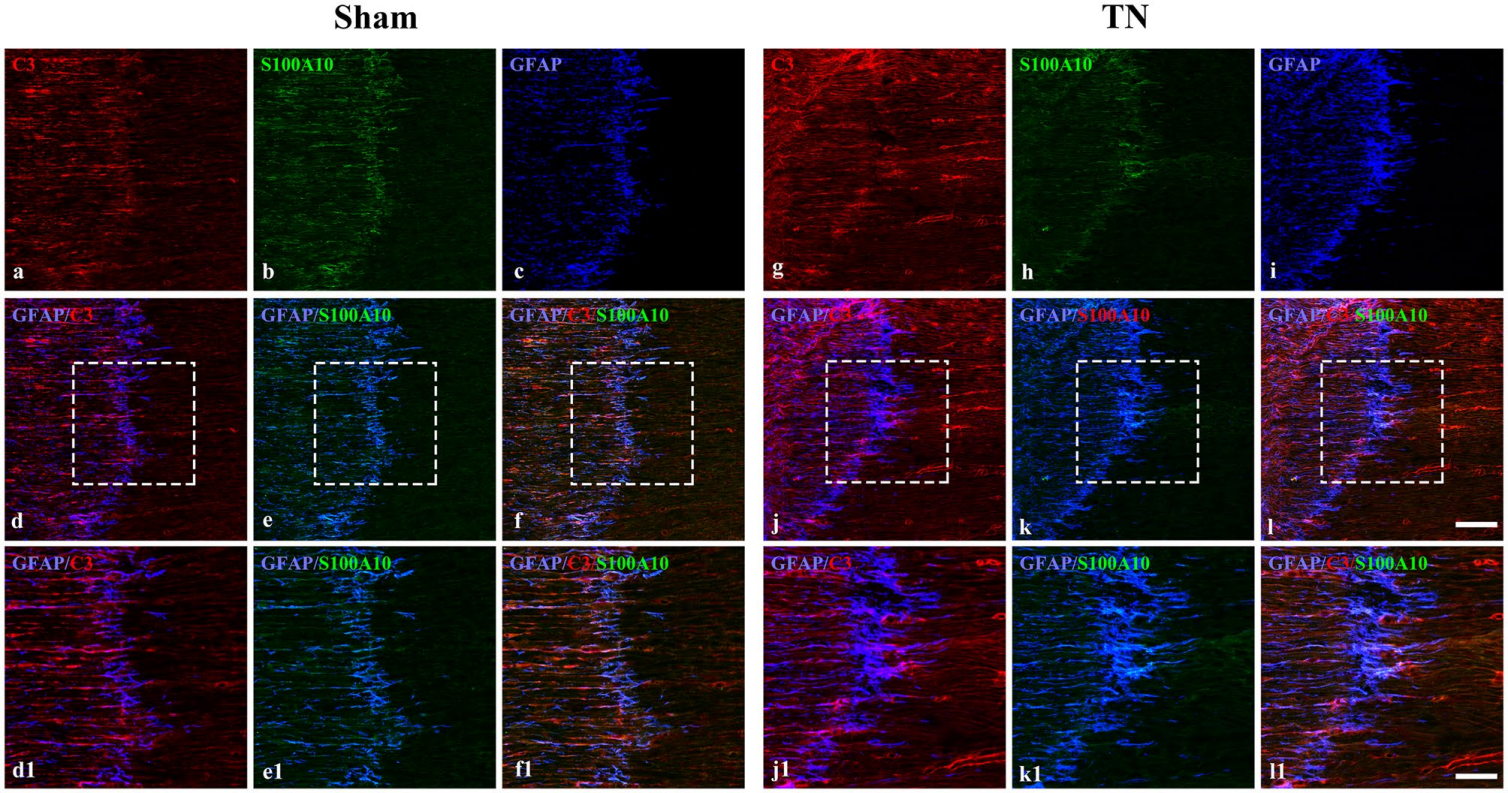
Immunofluorescence staining of A1- and A2-reactive astrocytes in the TREZ on POD 28. (**a**–**c**) and (**g**–**i**) Immunofluorescence staining of reactive astrocytes in the TREZ in the two groups. (**d**, **j**) show GFAP/C3-positive A1 astrocytes in the TREZ. (**c**, **k**) show GFAP/S100A10-positive A2 astrocytes in the TREZ. (**f**, **l**) show triple immunofluorescence staining of GFAP, C3 and S100A10. (**d1**–**f1**) and (**j1**–**l1**) show the higher magnification of (**d**–**f**) and (**j**–**l**). (**a**–**l**). scale bar = 100 μm. (**d1**–**f1**, **j1**–**l1**), scale bar = 50 μm.

**Figure 3:**
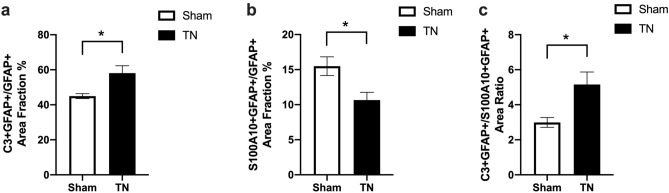
Quantitative analysis of C3 and S100A10 expression area in GFAP positive astrocytes in TREZ on POD 28. (**a**) Quantitative analysis showing significant up regulation of C3 in GFAP positive astrocytes on POD 28 in TN group compared to sham group. (**b**) Quantitative analysis showing significant lower expression of S100A10 in GFAP positive astrocytes on POD 28 in TN group compared to sham group. (**c**) The ratio of GFAP/C3-positive area and GFAP/S100A10-positive area was clearly increased in TN group compared with sham group. Data represent mean ± s.e.m. (n = 4–5 per group). *p < 0.05, as determined two-tailed Student’s t-tests.

On POD 28 of chronic compression, GFAP-positive astrocytes on the CNS side were significantly activated, thick protrusions of astrocytes extended obviously from the CNS to the PNS side, and GFAP expression had increased (Fig. 2). There were GFAP/C3-positive A1 astrocytes and GFAP/S100A10-positive A2 astrocytes on the CNS side of the TREZ region. More interestingly, some GFAP-positive reactive astrocytes expressed both C3 and S100A10 (Fig. 2 and Fig. S1). And there was significant larger expression area of the C3 (*p* = 0.0136) and obvious smaller expression area of the S100A10 (*p* = 0.0304) in GFAP positive astrocytes in TREZ after chronic compression. The ratio of GFAP/C3-positive area and GFAP/S100A10-positive area was clearly increased in TN group compared with sham group (*p* = 0.0174) (Fig. 3).

**Figure 4:**
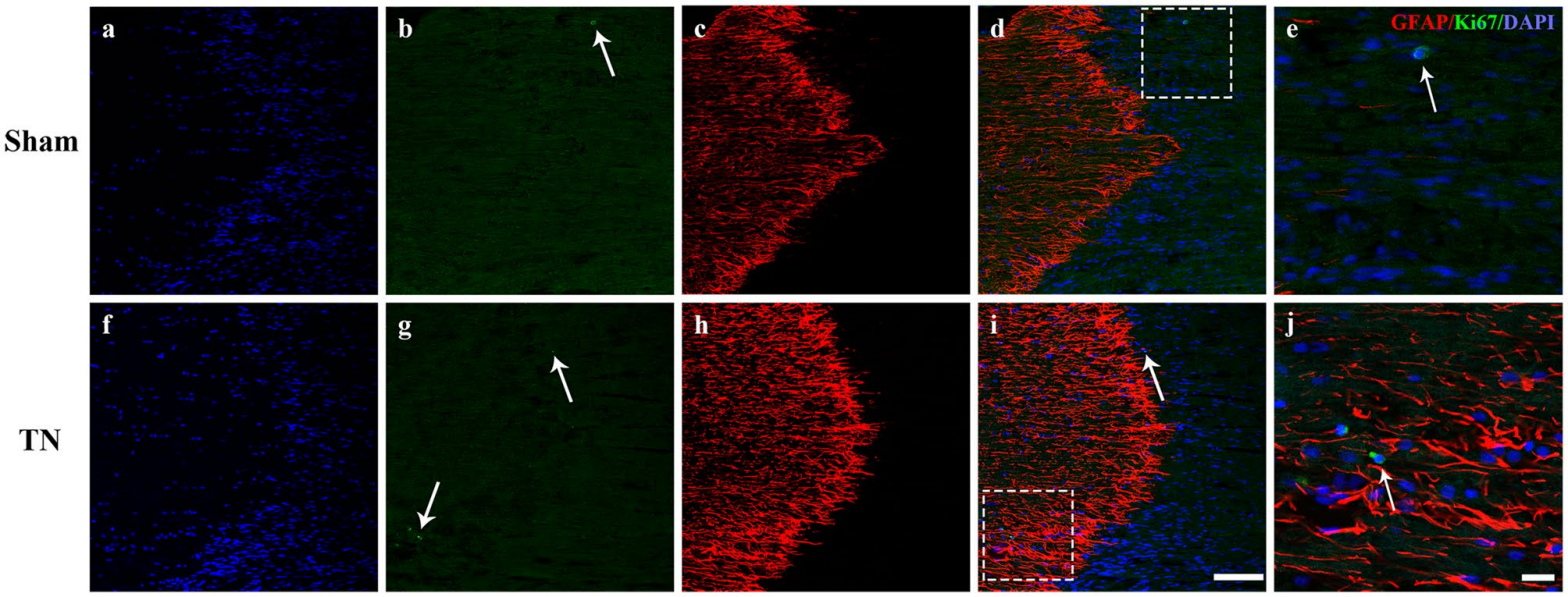
Immunofluorescence staining of GFAP, Ki67 and DAPI in the TREZ on POD 28. (**a**–**e**) and (**f**–**j**) show that the representative Ki67/DAPI-positive cells (white arrow) in the TREZ in two groups were both small, scale bar = 100 μm. (**e**) and (**j**) show the Ki67/DAPI-positive cells at higher magnification, scale bar = 25 μm.

**Figure 5:**
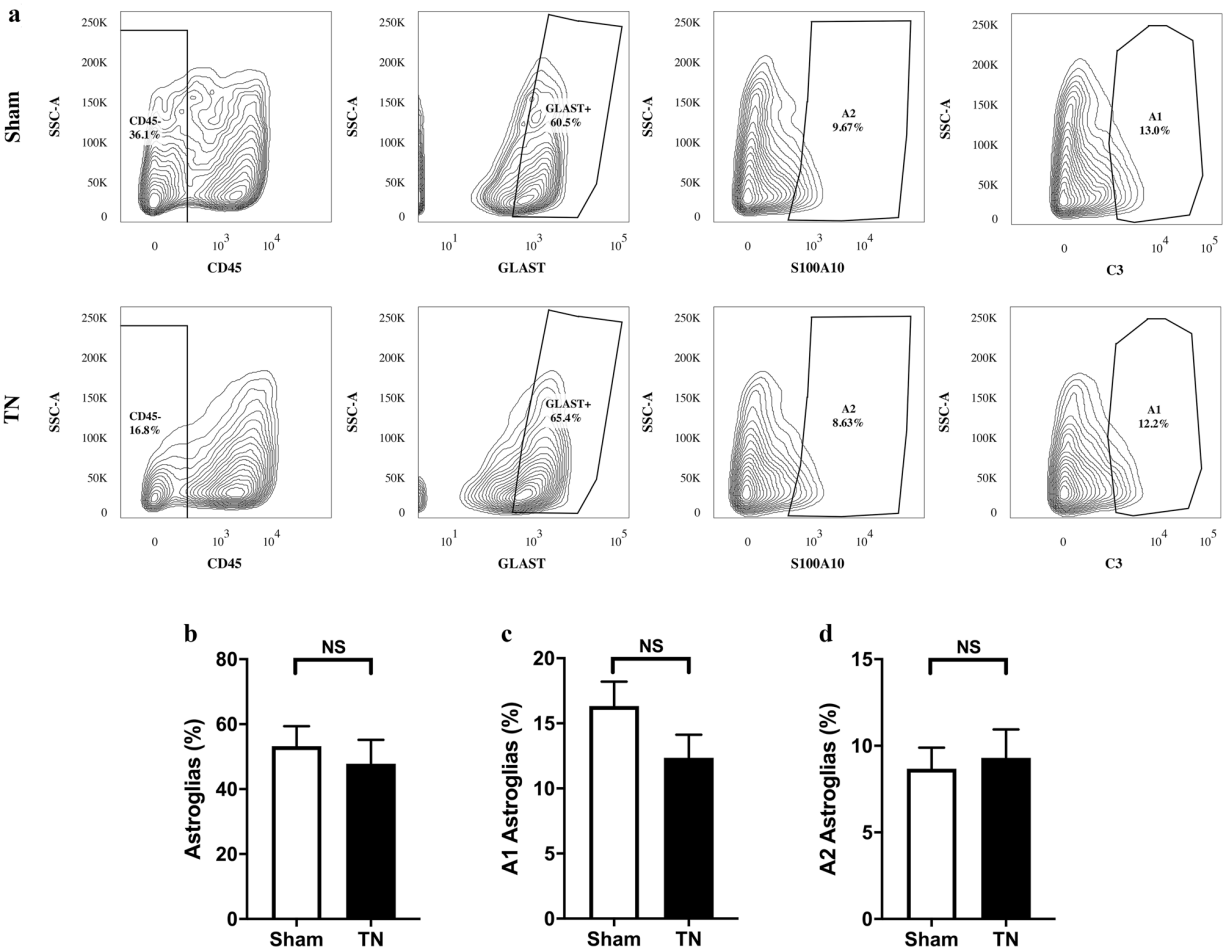
The proportions of different types of astrocytes in the TREZ and TG. (**a**) Flow contour plot of mature astrocytes (CD45^−^GLAST^+^), A1 astrocytes (CD45^−^GLAST^+^C3^+^) and A2 astrocytes (CD45^−^GLAST^+^S100A10^+^) in the TREZ and TG in the two groups of rats. (**b**–**d**) The difference between mature astrocytes (CD45^−^GLAST^+^) and A2 astrocytes (CD45^−^ GLAST^+^S100A10^+^) in the TN group and sham group was not statistically significant. Compared with the sham group, the proportion of A1 astrocytes (CD45^−^GLAST^+^C3^+^) in the TN group showed a downward trend (*p* = 0.1599). ns: not significant, as determined by two-tailed Student’s t-test.

However, there was very little positive expression of Ki67 in the TREZ region (Fig. 4). According to the result of flow cytometry, there were no statistical differences in the ratios of total astrocytes (CD45^−^GLAST^+^) (*p* = 0.05968), A1 astrocytes (CD45^−^GLAST^+^C3^+^) (*p* = 0.1599) and A2 astrocytes (CD45^−^GLAST^+^S100A10^+^) (*p* = 0.7788) between the two groups (Fig. 5).

### Different expression of immune cells in the TREZ after compression injury

**Figure 6:**
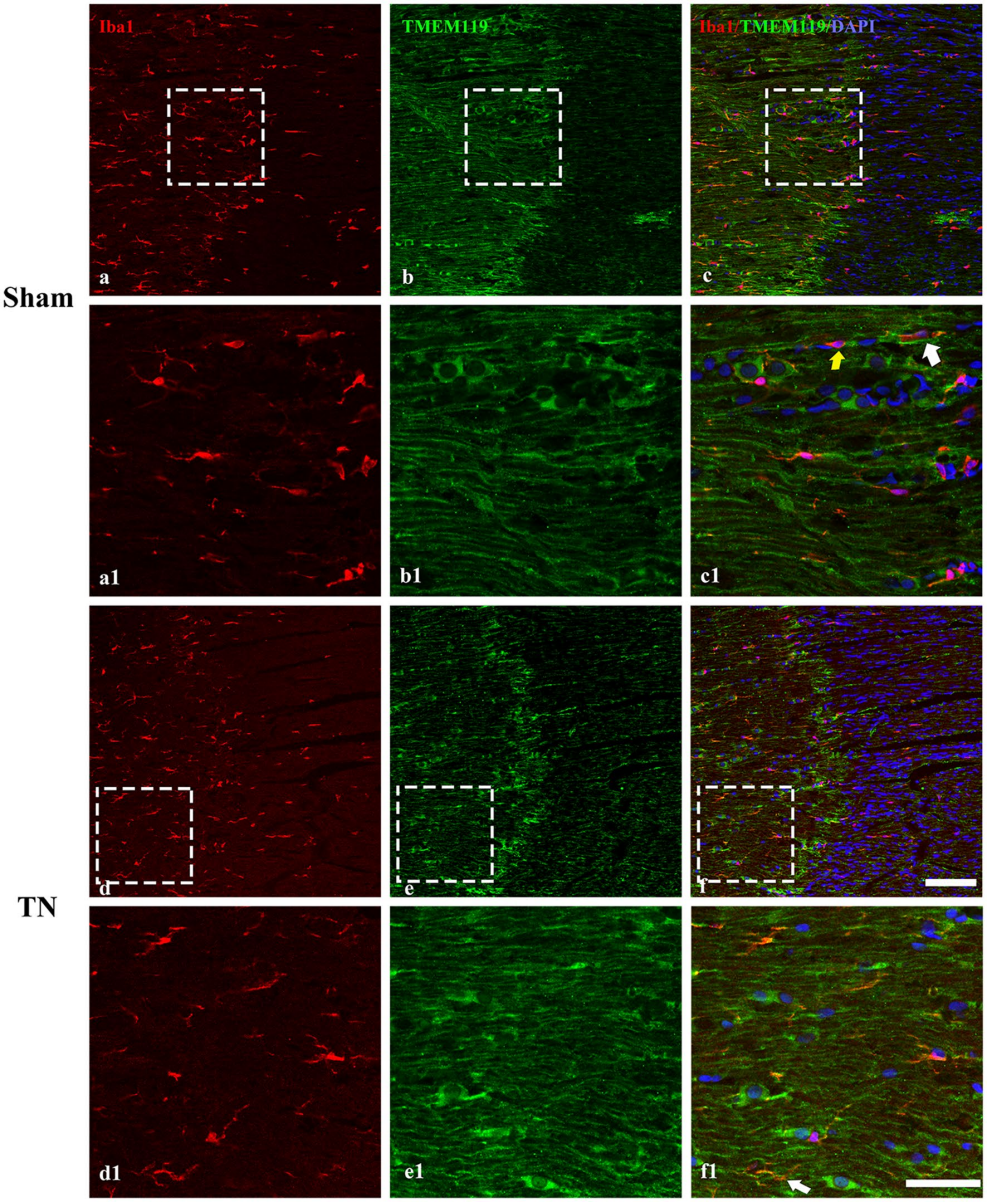
Immunofluorescence staining of Iba-1, TMEM119 and DAPI in the TREZ on POD 28. (**a**–**c**) and (**d**–**f**) show the expression of Iba-1 and TMEM119 in the TREZ in the sham group and the TN group; scale bar = 100 μm. (**a1**–**c1**) and (**d1**–**f1**) show the Iba-1^+^/TMEM119^+^ cells (white arrow) and Iba-1^+^/TMEM119^−^ cells (yellow arrow) in the TREZ, and there were also exited Iba-1^−^/TMEM119^+^ cells; scale bar = 50 μm.

According to the immunohistochemical staining results, there was no significant difference in Iba-1-positive cells between the two groups on POD 28, and the cell marker TMEM119 was present not only in Iba-1-positive cells but also some other cells (Fig. 6).

**Figure 7:**
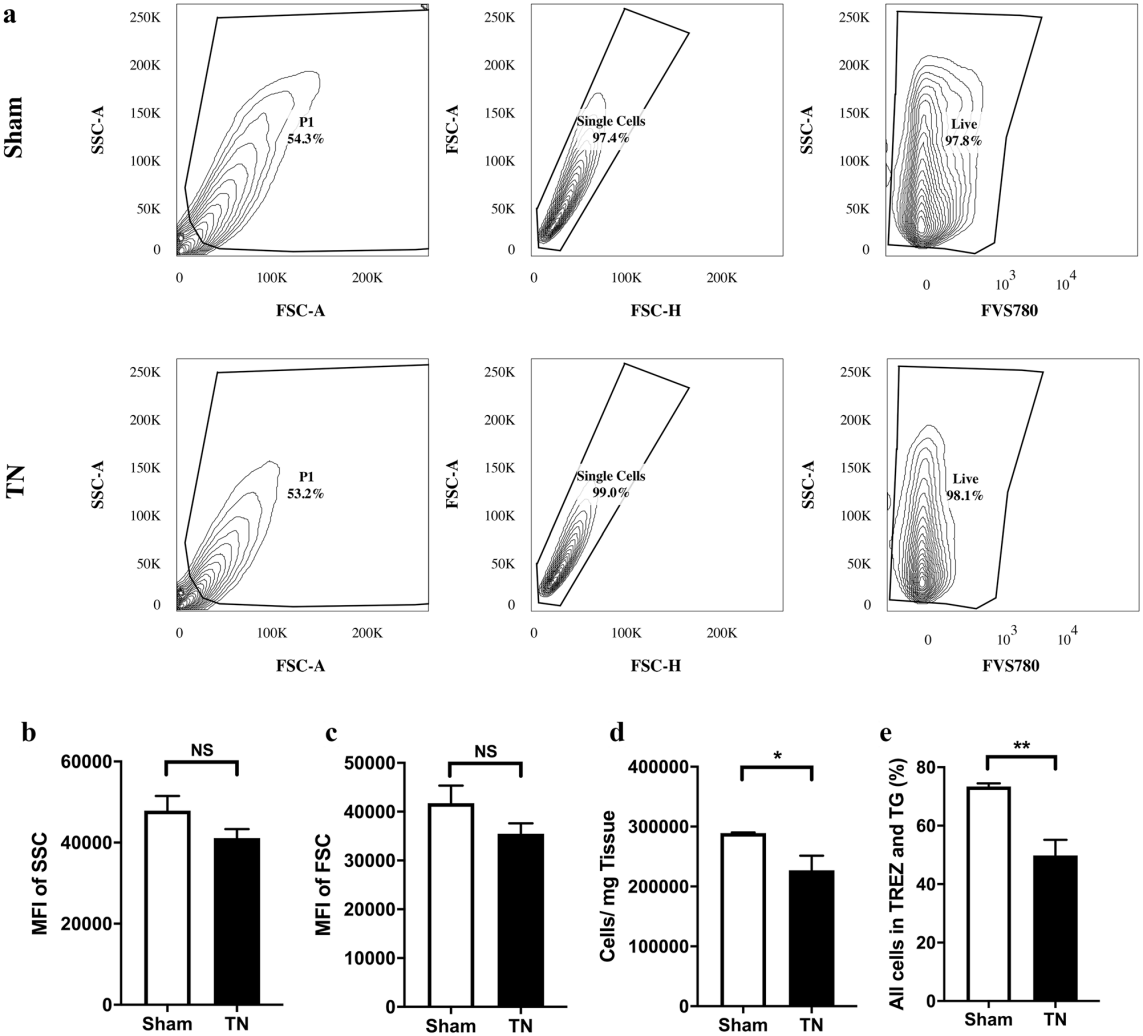
Cell size, granularity and relative cell number and proportion in the TREZ and TG. (**a**) Most cells were alive when analyzed in a BD LSR Fortessa X-20. (**b**, **c**) The relative size and granularity of all cells in the TREZ and TG in the two groups were not significantly different. (**d**, **e**) The total cell number and percentage of cells per milligram of tissue in the TN group were significantly less than those in the sham group. *p < 0.05, **p < 0.01, ns: not significant, as determined by two-tailed Student’s t-test. *FSC* forward scatter, *SSC* side scatter, *PI* propidium iodide.

The survival rates of all cells extracted from both groups were calculated to be above 95% on POD 28 by flow cytometry. The relative cell size and the cell granularity in the two groups were not measurably different. Quantification analysis was performed by collecting cells with a Trucount™ absolute counter, and the absolute number of cells per milligram of tissue was calculated. According to the total cell number of sham group (289,182 ± 1166) vs. TN group (227,194 ± 24,227) (*p* = 0.00443), and percentage of sham group (73.42 ± 1.044%) vs. TN group (49.82 ± 5.43%) of cells per milligram of tissue (*p* = 0.0034), the total cell number and percentage in TN group were significantly less than those in the sham group (Fig. 7).

**Figure 8:**
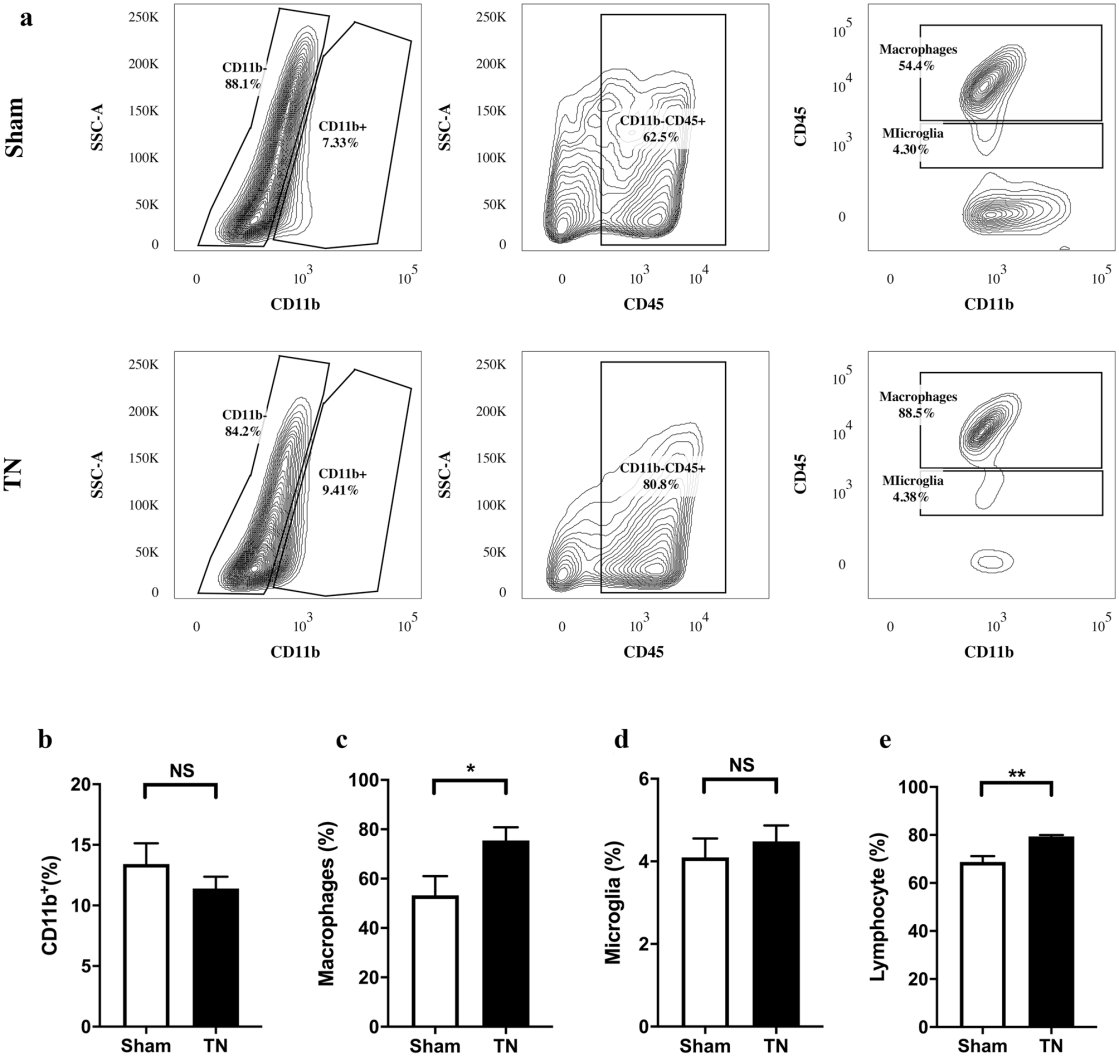
Numbers and proportions of immune-related cells in the TREZ and TG. (**a**) Flow contour plot of immune-related cells in the TREZ and TG in the two groups of rats. (**b**) The ratios of CD11b^+^ cells in the TREZ and TG of the TN group were lower than those of the sham group. (**c**–**e**) The ratios of macrophages (CD45^high^CD11b^+^) and lymphocytes (CD45^+^CD11b^−^) in the TREZ and TG of the TN group were higher than those in the sham group. The difference in the proportions and numbers of microglia (CD45^low^CD11b^+^) between the two groups was not statistically significant. *p < 0.05, **p < 0.01, ns: not significant, as determined by two-tailed Student’s t-test. *SSC* side scatter.

The proportions and numbers of CD11b^+^ cells in the TREZ and TG in the two groups were not significantly different (*p* = 0.3327) (Fig. 8). The ratios of infiltrating lymphocytes (CD45^+^CD11b^−^) (*p* = 0.0075) and infiltrating macrophages (CD45^high^CD11b^+^) (*p* = 0.0388) were significantly higher than those of the sham group. There was no significant difference in the ratio and number of microglia (CD45^low^CD11b^+^) between the two groups (*p* = 0.5304) (Fig. 8).

## Discussion

In this study, the orofacial mechanical pain threshold tested by von Frey filaments is to confirm the successful establishment of TN animal model, and the histological analyses have been well studied in our previous researches^4,13,17^. We consider that it is one kind of the relatively stable and mature TN animal models induced by mechanical compression on the TREZ.

Based on the immunohistochemical staining results, chronic compression of TREZ induced an increase in GFAP expression in astrocytes, and the protrusions of astrocytes became thicker and obviously extended to the peripheral side (Fig. 2 and Fig. S1). The co-staining of the GFAP/C3-positive A1-astrocytes obviously activated after chronic compression injury in TN group, while the expression of GFAP/S100A10-positive A2-astrocytes activation was relatively low. Interestingly, some triple-labeled GFAP/C3/S100A10-positive cells were also observed in the TREZ of the TN rat model, while few Ki67-positive cells were observed (Fig. 4). Flow cytometry analysis also showed that the ratios of A1 astrocytes (CD45^−^GLAST^+^C3^−^) and A2 astrocytes (CD45^−^GLAST^+^S100A10^+^) did not change significantly. GLAST antibody is a specific marker which is widely used in astrocytes flow cytometry and sorting, as the previous literature described ^18^. Besides, we focus on the activation of astrocytes on post operation day 28 in the TN animal model, the inflammation in the rats may be largely alleviated at this time. Therefore, we consider that the isolation efficacy of astrocytes by using GLAST in our study might not affect the results obviously. We considered that morphological and functional changes, rather than the proliferation of astrocytes, may be more susceptible to the influence of compression injury on the TREZ of TN rats.

Flow cytometry was used to distinguish clearly and effectively between microglia (CD45^low^CD11b^−^) and macrophages (CD45^high^CD11b^−^) in our study. The ratios of infiltrating macrophages (CD45^high^CD11b^+^) and lymphocytes (CD45^+^CD11b^−^) increased, while the ratio of microglia (CD45^low^CD11b^+^) remained unchanged, indicating that infiltrating immune cells were more common than proliferating innate immune cells in the TREZ and TG in the process of chronic compression injury. Infiltrating macrophages (CD45^high^CD11b^+^) and lymphocytes (CD45^+^CD11b^−^) may play important roles in the occurrence and development of TN induced by chronic compression. CD11b is commonly used to identify cells in myeloid lineage, including neutrophils, monocytes, macrophages and microglia. Although the ratio of total CD11b-positive cells in the TREZ was reduced a little in our study, while there was no significant difference between sham group and TN group (Fig. 8). We think it may be the reason that compression injury on TREZ leads to the decrease of absolute cell numbers in nerve tissue samples obtained. Furthermore, chronic mechanical compression on the TREZ in TN model could also undermine the integrity of blood–nerve-barrier inside the trigeminal nerve causing the macrophages infiltrate to the parenchyma from blood–nerve-barrier, which may increase the ratio of macrophages.

Recent studies have shown that the complex interconnections between glial cells and immune cells play a crucial role in the maintenance of CNS homeostasis, inflammation, and neuropathic pain^19^. Astrocytes reversibly regulate the demyelination and remyelination of myelinated nerve fibers during nerve injury by secreting different cytokines^20^. CCL2 and CXCL10, which are produced by reactive astrocytes, can recruit lymphocytes in the peripheral blood to the CNS, and astrocytes provide nutritional support for these infiltrating immune cells^21^. Infiltrating macrophages and lymphocytes may migrate through the broken blood–nerve barrier in chronic compression nerve injury with the action of certain chemokines and participate in the occurrence and development of TN induced by chronic compression.

Activated microglia dominates the early glial response to peripheral nerve injury and participate in the induction and development of neuropathic pain, while astrocytes contribute to the persist of chronic pain^22,23^. In our previous study, Iba1-positive microglia/macrophages increased on POD 7 and POD 14 in the TREZ of TN rats, and these activated cells decreased to POD 21 and returned to a low level on POD 28^4^. Therefore, the results of the change in cell number by flow cytometry showed no change in microglia on POD 28, which is also consistent with our previous study.

However, our experiments also had some limitations. Although TMEM119 has been identified as a surface marker of microglia that can be used to reliably distinguish microglia from infiltrating macrophages^24^, recent studies also reported that the expression of TMEM119 is not restricted to microglia in inflammation^25,26^. In our study, there were few Iba1/TMEM119-positive cells in the TREZ, so we still could not well distinguish between microglia and macrophages through the two cell markers by immunohistochemistry.

In summary, chronic compression of the TREZ induced orofacial mechanical hyperalgesia in TN rats, A1-astrocytes rather than A2-astrocytes obviously activated after chronic compression injury in TN group, while infiltrating macrophages and lymphocytes increased, which indicating the neuroimmune cells in the TREZ may participate in the pathogenesis of TN rat model.

## Supplementary Information

Supplementary Figure S1.

## Abbreviations

TN: Trigeminal neuralgia
TREZ: Trigeminal root entry zone
TG: Trigeminal ganglion
CNS: Central nervous system
PNS: Peripheral nervous system
GLAST: Glutamate-aspartate transporter
