# Supplementary material for: Flow cytometry analysis of immune and glial cells in a trigeminal neuralgia rat model
Source: Sci Rep. 2021 Dec 7;11:23569. doi: 10.1038/s41598-021-02911-x (PMC8651642; doi:10.1038/s41598-021-02911-x)
Supplement: Supplementary file 1 — Supplementary Figure S1. [file 41598_2021_2911_MOESM1_ESM.docx]

**Supplementary Information**

**Flow cytometry analysis of immune and glial cells in a trigeminal neuralgia rat model**

Junjin Lin^1#^，Luxi Zhou^2,3#^，Zhaoke Luo^2,3^，Madeha Ishag Adam^2,3^，Li Zhao^2,3^，Feng Wang^1,2,3^*，Daoshu Luo^1,2,3^*

1. Public Technology Service Center of Fujian Medical University; Laboratory of Clinical Applied Anatomy, School of Basic Medical Sciences, Fujian Medical University, Fuzhou 350122

2. Key Laboratory of Brain Aging and Neurodegenerative Diseases of Fujian Province, Fuzhou, 350122, China.

3. Department of Human Anatomy, School of Basic Medical Sciences, Fujian Medical University, Fuzhou 350122

# Junjin Lin and Luxi Zhou contributed equally to this work.


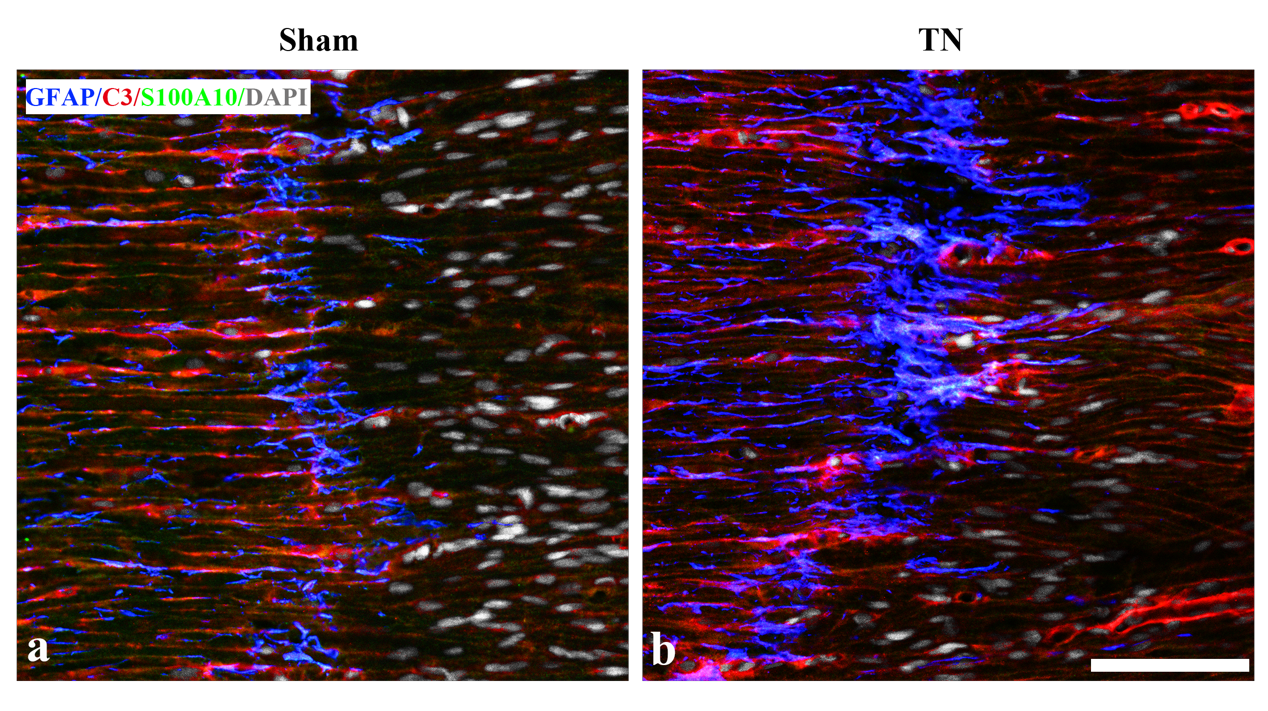


**Figure S1**: Immunofluorescence staining of GFAP, C3, S100A10 and DAPI in the TREZ on POD 28. GFAP-immunoreactive astrocytes (pseudo-color blue) were shown in the TREZ. C3 (pseudo-color red) were expressed in many cells including GFAP positive astrocytes. S100A10 (pseudo-color green) were expressed mainly in GFAP positive astrocytes. Nuclei were stained with DAPI (pseudo-color gray). **(a)** show GFAP/C3-positive A1 astrocytes (purple) and GFAP/S100A10-positive A2 astrocytes (cyan) in the TREZ in sham group. **(b)** show GFAP/C3-positive A1 astrocytes (purple) and GFAP/S100A10-positive A2 astrocytes (cyan) in TN group. Scale bar = 50 μm.
